# Different cellular response mechanisms contribute to the length-dependent cytotoxicity of multi-walled carbon nanotubes

**DOI:** 10.1186/1556-276X-7-361

**Published:** 2012-07-02

**Authors:** Dun Liu, Lijun Wang, Zhigang Wang, Alfred Cuschieri

**Affiliations:** 1 Institute for Medical Science and Technology (IMSaT), University of Dundee, Wilson House, 1 Wurzburg Loan, Dundee Medipark, Dundee DD2 1FD, UK

**Keywords:** Carbon nanotube, Cytotoxicity, Length effect, Cell type

## Abstract

To date, there has not been an agreement on the best methods for the characterisation of multi-walled carbon nanotube (MWCNT) toxicity. The length of MWCNTs has been identified as a factor in *in vitro* and *in vivo* studies, in addition to their purity and biocompatible coating. Another unresolved issue relates to the variable toxicity of MWCNTs on different cell types. The present study addressed the effects of MWCNTs' length on mammalian immune and epithelial cancer cells RAW264.7 and MCF-7, respectively. Our data confirm that MWCNTs induce cytotoxicity in a length- and cell type-dependent manner. Whereas, longer (3 to 14 μm) MWCNTs exert high toxicity, especially to RAW264.7 cells, shorter (1.5 μm) MWCNTs are significantly less cytotoxic. These findings confirm that the degree of biocompatibility of MWCNTs is closely related to their length and that immune cells appear to be more susceptible to damage by MWCNTs. Our study also indicates that MWCNT nanotoxicity should be analysed for various components of cellular response, and cytotoxicity data should be validated by the use of more than one assay system. Results from chromogenic-based assays should be confirmed by trypan blue exclusion.

## Background

Since the landmark paper on carbon nanotubes (CNTs) by Iijima in 1991 [1], extensive research has confirmed their unique physical, chemical, and electrical properties generating considerable interest in their potential biomedical applications, e.g. drug delivery [2], tumour hyperthermic ablation [3], and tissue engineering [4]. As with any potential therapeutic or diagnostic agents, the safety profile (biocompatibility and potential adverse effects) of CNTs is crucial for the translation into novel clinical therapies based on these nanomaterials. Several *in vitro* and *in vivo* studies have demonstrated that short, highly purified CNTs with biocompatible polymer coating and functionalisation are essential for reduction in cytotoxicity [5-8]. However, for various reasons, there remain conflicting data concerning CNT toxicity: lack of characterisation of the CNTs and differences in the experimental design, materials, cell viability systems, and nanotube concentrations.

In the assessment of cytotoxicity, cell types also need to be considered as cells with different functions may respond differently to the exposure of CNTs. After the CNTs' pass of the endothelial barrier, the cell types that are most likely to encounter CNTs *in vivo* are immune cells (e.g. macrophages, dendritic cells, and T and B lymphocytes) because of their primary defence function against pathogens and foreign particulate matter. Additionally, immune cells have been identified as effective vehicles for cancer therapy and in the treatment of infectious diseases when stimulated and targeted by functionalized CNTs [9-11]. Hence evaluation of CNT toxicity to immune cells is an important but overlooked component of CNT nanotoxicology.

In the present study, we investigated the length-dependent effects of multi-walled carbon nanotubes (MWCNTs) *in vitro* on two types of cell lines: macrophage-like RAW264.7 and epithelial cancer cell line MCF-7, using highly purified and stable aqueous dispersed MWCNTs that were characterised by scanning electron microscopy (SEM). We confirm that long MWCNTs are more toxic to both cell types and that the toxicity of MWCNTs is greater to RAW264.7 cells than it is to MCF-7 cells. Possible mechanisms for the differences in the degree of cytotoxicity were investigated by measuring MWCNT cellular uptake, reactive oxygen species (ROS) generation, and inflammatory responses of RAW264.7 cells by cytokine secretion. Our data indicated that nanotoxicity analysis requires a different approach to conventional toxicity studies used for cytotoxic drugs, and for valid assessment of the effect of CNTs on cell viability, different assay systems that measure different aspects of cellular response should be used.

## Methods

### Preparations of MWCNT solution

Pure MWCNTs (99 %) of the same diameter (40 to 60 nm) but in two length ranges: 1.5 (short (S)-MWCNTs) and 3 to 14 μm (long (L)-MWCNTs) were supplied by Nanothinx S.A. (Platani, Greece) produced by chemical vapour deposition. MWCNT purity was estimated by ICP-MS, which accounts for the percentage of metal residuals. The aqueous dispersions of MWCNTs were realized by coating with non-ionic surfactant Pluronic F-127 (polyoxyethylene-polyoxypropylene block copolymer, PF-127; Sigma-Aldrich, Dorset, UK, cat# P2443), which has been proved to be successful dispersion agent for CNTs [12]. The coating was achieved by mixing 40-mg MWCNTs with 80 mL of a PF-127 solution (1 % PF-127 in PBS). This solution was stirred at 70 °C for 5 h and then sonicated at 70 W (Branson sonicator, Bransonic, Danbury, CT, USA) for 2 h to achieve homogeneous dispersion. The dispersion was then centrifuged at 1,100 × *g* for 10 min to remove residuals and impurities. Supernatant was collected, and PF-127-coated MWCNTs were washed three times at 40,000 g, 4 °C for 30 min with Dulbecco's modified Eagle's medium (DMEM) containing 10 % fetal calf serum (FCS) and penicillin/streptomycin, to remove the excess PF-127 in solution. The residual pellet was re-dispersed in DMEM to form the final solution at a concentration of 800-μg/mL MWCNTs before storage at 4 °C. The PF-127 coating was confirmed by SEM [see Figure S1 in Additional file 1. The concentrations of MWCNT solutions were measured by spectrophotometric analysis [13].

### Characterisation of MWCNTs by SEM

Scanning electron microscopy was employed to assess effective dispersion of both S- and L-MWCNTs and also to confirm the size distribution of MWCNTs of the two samples. For this characterisation, samples of polymer-coated S- and L-MWCNTs were washed eight times with ddH_2_O by centrifugation at 40,000 × *g*, 4 °C, to remove the salts and proteins in the medium. A small drop (10 μL) of MWCNT solution (5 μg/mL) was placed onto an alumina substrate and allowed to dry at room temperature. Dried specimens were then coated with 8-nm Au/Pd using a Cressington 208HR sputter coater (Cressington Scientific Instruments Inc., England, UK). Specimens were examined using a Philips XL30 ESEM (FEI Co., Hillsboro, OR, USA) operating at an accelerating voltage of 15 kV.

### Cell culture

Two cell lines were used for this study: phagocytic immune cell line - murine macrophage cells RAW264.7, kind gift from Professor Colin Watts and Dr. Alan Prescott (College of Life Sciences, University of Dundee), and non-phagocytic epithelial cell line - human breast cancer MCF-7 cells (ATCC; cat# CCL-228). RAW264.7 and MCF-7 cells were grown in DMEM (GIBCO, Invitrogen, Paisley, UK). Media were supplemented with 10 % FCS, 2-mM glutamine, 100-IU/mL penicillin, and 100-μg/mL streptomycin. Cells were grown under standard cell culture conditions in 5 % CO_2_ at 37 °C to reach confluence of 50 % to 60 % before subjected to any further treatment.

### Analysis of cellular uptake of MWCNTs by TEM

Cells were grown in 75-cm^2^ flasks in standard conditions described above. Culture medium was then changed to one containing MWCNTs at concentration of 50 μg/mL, and cells were incubated for further 24 h. Cells were washed and fixed with 4 % (*w*/*v*) paraformaldehyde/2.5 % (*v*/*v*) glutaraldehyde buffered in 0.2-M PIPES. After 30 min in fixative, cells were scraped off the flasks in 1 ml of fixative, transferred to Eppendorf tubes, and centrifuged at 14,000 × *g* for 20 min. Pellets were washed in 0.2-M PIPES and postfixed in 1 % aqueous osmium tetraoxide for 1 h, washed in water, and dehydrated in graded ethanol and propylene oxide before embedding in Durcupan resin (Sigma) and polymerised at 60 °C for 24 h. Seventy-nanometer sections were cut on a Leica UCT ultramicrotome (Leica Microsystems Ltd., Milton Keynes, UK), mounted on Pioloform-coated 100-mesh copper grids, stained with uranyl acetate and lead citrate before being examined in a Tecnai 12 electron microscope (E.A. Fischione Instruments, Inc., PA, USA). Images were recorded in digital imaging plates and scanned in a Ditabis Micron scanner (Pforzheim, Germany).

### Quantitative measurement of cellular uptake of MWCNTs

Cells were seeded in 6-well plates and exposed to 10 μg/mL S- and L-MWCNTs for 0.5, 1, 4, 8, and 24 h. At each time intervals, cells were washed with PBS three times and trypsinized for counting. Cells were counted, and same number of cells (10^5^ cells) were centrifuged and lyzed with 800-μL 2 % sodium dodecyl sulfate solution at room temperature for 10 min. Dimethyl sulfoxide (200 μL) was then added to the lysate to facilitate MWCNT dispersion. Lysate (100 μL) was transferred to a 96-well plate, and absorbance at 500 nm was measured using multimode plate reader (infinite M200, Tecan Austria GmbH, Salzburg, Austria). MWCNT concentrations were calculated according to the initially established standard MWCNT solution curve. The method is based on the work by Hirano et al. [14].

### Cell viability assays

Two cell viability assays were used to study the effect of MWCNT treatment. The first was the CellTiter-Blue Cell Viability Assay (Promega, UK). In this assay, viable cells convert a non-fluorescent compound resazurin into fluorescent end product resorufin. Cells were seeded in 96-well plates at densities of 10 × 10^4^, 5 × 10^4^, 2.5 × 10^4^, and 1.25 × 10^4^ cells/mL, respectively, depending on the incubation time required and maintained in cultures for 24 h before addition of MWCNTs. MWCNTs in media were diluted by culture media to final concentrations of 200, 50, and 12.5 μg/mL and added to cell cultures (in triplicate), respectively. Cells were incubated with MWCNTs for up to 1 week. Cells incubated with MWCNTs at each time interval were washed (three times) with culture media to remove the excessive MWCNTs before addition of assay reagents. Cells were further incubated for 3 h, and fluorescence intensity was measured using wavelengths of excitation at 560 nm and emission at 590 nm, respectively. Values (fluorescence intensity at 590 nm) of treated cells were expressed as percentage of that from corresponding control cells. All experiments were repeated at least three times.

Trypan blue exclusion was used as the second assay to validate cell viability data obtained by the CellTiter-Blue method. Trypan blue is a vital stain used to colour the dying or dead cells in which cell membranes lose their integrity so the dye can pass through the membrane and stain the cell. In contrast, healthy, viable cells maintain their membrane integrity and cannot be stained by trypan blue. Cells were cultured and treated the same way as described in the CellTiter-Blue assay except seeded in 24-well plates in duplicate. Cells incubated with MWCNTs at each time interval were washed twice with PBS before trypsinization, following which cell suspensions were mixed with equal volume of 0.4 % trypan blue. Then 10 μL of stained cells was placed in an automated cell counter (Countess automated cell counter, Invitrogen, UK), and the number of viable cells was counted.

### ELISA measurements of cytokine secretion of RAW264.7

RAW264.7 cells were treated by S- and L-MWCNTs at concentrations of 12.5, 50, and 200 μg/mL, respectively, for 24 h, and the supernatants of culture media were assayed for TNF-α and interleukin-12 (IL-12) concentrations by ELISA using commercially available mouse TNF-α kit (Invitrogen, UK; cat# KMC3011) and mouse IL-12 (p70) ELISA MAX Deluxe Set (BioLegend, UK; cat# 433604), respectively, according to the manufacturer's instructions. In brief, cell culture supernatants were added to cytokine specific antibody-coated wells, followed by the addition of a biotinylated detection antibody. Avidin-horseradish peroxidase conjugate was then added to plate wells followed by addition of appropriate enzyme substrate. The resultant absorbance of samples was quantitated at 450 nm using multimode plate reader (infinite M200, TECAN, Austria), and background was subtracted. Experiments were conducted in triplicate for three times.

### Measurement of intracellular ROS

ROS generation by MCF7 and RAW264.7 was determined by ROS assay according to the manufacturer's instructions. In brief, RAW264.7 and MCF-7 cells were seeded in 96-well plates at a density of 10^5^ cells/well and incubated at 37 °C for 24 h. Culture media were aspirated, and cells were treated by S- and L-MWCNT solutions at concentrations of 12.5, 50 and, 200 μg/mL, respectively, for 24 h. After treatment, cells were washed with PBS, loaded with 10-μM carboxy-H_2_DCFDA (Invitrogen, UK; cat# C6827) for 1 h. Subsequently, loading buffer was removed, and cells were returned to pre-warmed media and incubated for 30 min before the final reading. In the final step, media were replaced by PBS, and fluorescence intensities were measured at 493-nm excitation and 520-nm emission in the multimode plate reader. Carboxy-H_2_DCFDA unstained cells with MWCNTs for autofluorescence measurements in order to subtract the background reading were assessed as negative controls. Experiments were carried out in duplicate and repeated three times.

### Statistical analysis

Results were expressed as mean ± standard errors. *P* values for differences between MWCNT treated samples and controls were calculated using unpaired two-tailed equal variance Student's *t*-test. Statistical significance was set at *p* < 0.05.

## Results

### Characterisation of MWCNTs by SEM

**Figure 1:**
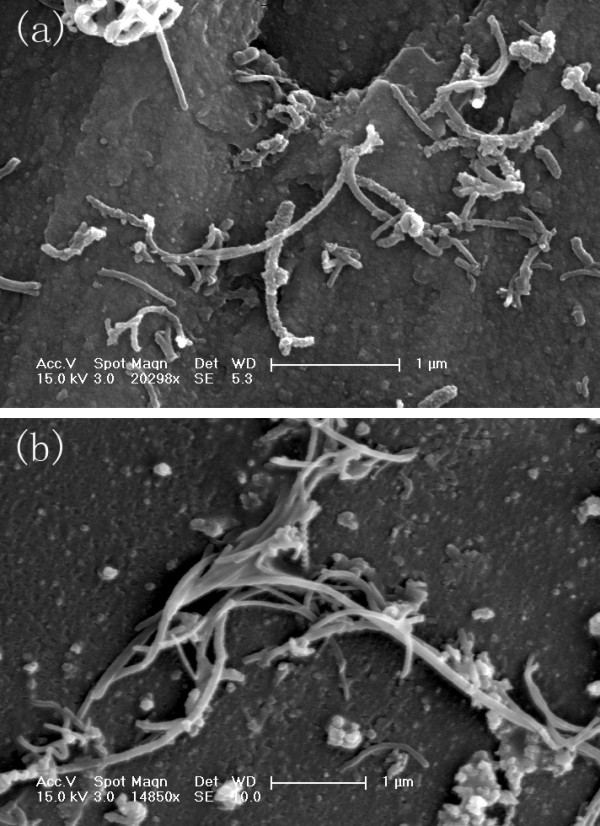
SEM images of (a) S- and (b) L-MWCNTs.

The aqueous dispersions of MWCNTs were obtained by coating with non-ionic surfactant PF-127 and were stable for at least 6 months after coating (data not shown). Metal content remained in MWCNTs was less than 0.2 %, verified by ICP-MS (data not shown). After washing with ddH_2_O, S- and L-MWCNTs were examined by SEM. SEM images in Figure 1 show that more entanglements occurred among L-MWCNTs than S-MWCNTs (Figure 1a,b). By manually counting 50 of each of the two types of MWCNTs, S-MWCNTs were of 0.4 to 1.4 μm in length as were claimed by the manufacturer (<1.5 μm), and L-MWCNTs were in the range of 2.4 to 10 μm with most L-MWCNTs in 3 to 8 μm, which are slightly shorter than the manufacturer claimed (3 to 14 μm) but have no overlap with S-MWCNTs. We believe that the length difference between these two MWCNT samples was large enough for the length comparison study, and the possible effects caused by metal content can be neglected.

### Cellular uptake of MWCNTs

**Figure 2:**
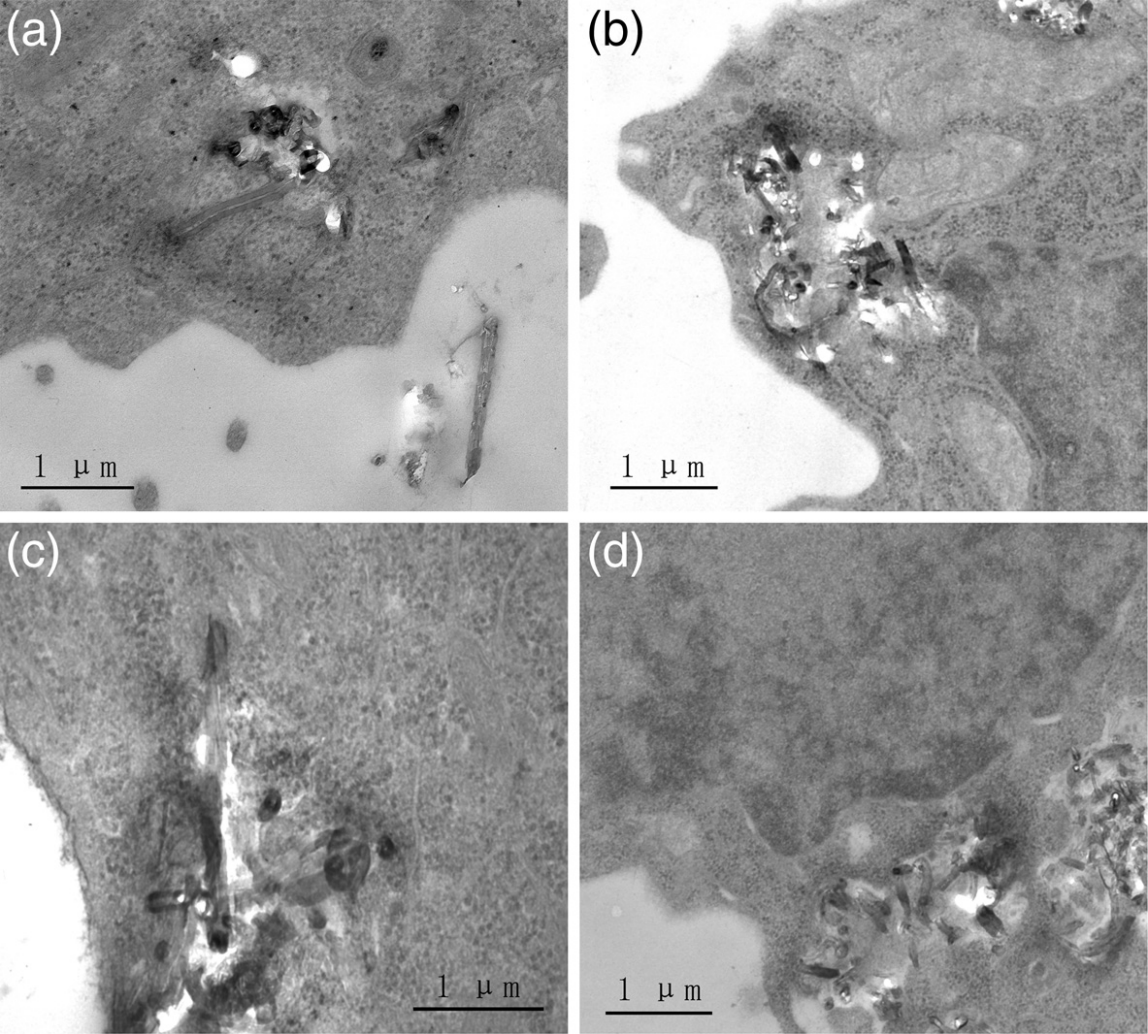
**TEM photographs show the cellular uptake of MWCNTs.** Cells were incubated overnight in media containing 50-μg/mL S- (**a** and **b**) and L-MWCNTs (**c** and **d**), and MWCNTs internalisation in MCF-7 (a and c) and RAW264.7 cells (b and d) were analysed by TEM as described in the ‘Methods’ section.

**Figure 3:**
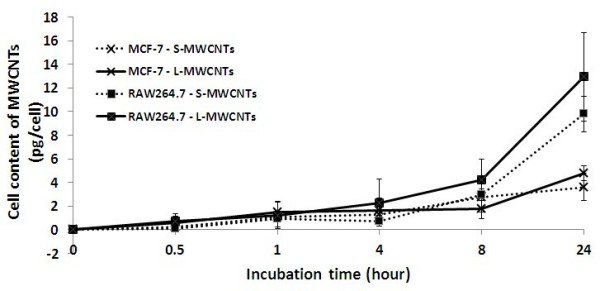
**Cellular uptake of MWCNTs.** Cells were incubated in media containing 10-μg/mL S- and L-MWCNTs for up to 24 h. Absorbance was taken after the cells were thoroughly washed (three times) and lysed as described in the ‘Methods’ section. Experiments were performed in triplicate for three times.

The mechanism of CNT cellular uptake has yet to be fully understood but may occur through either an energy-independent needle-like penetration, or engulfment, or a combination of both processes, possibly depending on CNT surface characteristics and cell types [3,15]. To confirm the internalisation of MWCNTs, cellular uptake of S- and L-MWCNTs was examined by TEM. Figure 2a,b shows that S-MWCNTs were identified in the cytoplasm of MCF-7 and RAW264.7 cells. Similarly, cellular uptake of L-MWCNTs by both cells was also observed by TEM (Figure 2c,d). These results confirmed the internalisation of MWCNTs by both epithelial tumour and phagocytic immune cells. To quantitatively measure the amount of MWCNTs that interacted with cells including intracellular and those attached to cell membranes, spectrophotometry was performed, and the data revealed that RAW264.7 cells had significantly more S-MWCNTs internalised or attached than the epithelial cells after 24-h incubation (*p* < 0.05) (Figure 3). Concentration of 10 μg/mL was chosen because it caused minimal viability loss to all cell lines after 24-h incubation based on the viability data (see below). As shown in Figure 3, RAW264.7 again exhibited higher uptake of L-MWCNTs than MCF-7. RAW264.7 cells had significant higher uptake of L-MWCNTs over MCF-7 cells after 24 h. By checking under optical microscope, we observed more S- and L-MWCNTs were taken or membrane bounded by RAW264.7 cells than MCF-7 cells (data not shown). Difference in MWCNT uptake may affect cell viability and functionality to different extent, and further investigations are needed to clarify this.

### Cell viability measured by CellTiter-Blue and trypan blue staining

**Figure 4:**
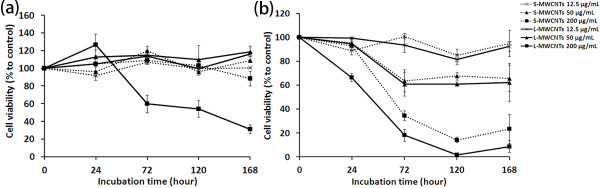
**Cell viability of RAW264.7 cells.** Measured by (**a**) CellTiter-Blue assay and (**b**) trypan blue counting in response to different concentrations and incubation times of S- and L-MWCNTs. Cells were treated as described in the ‘Methods’ section, and data were expressed as mean ± standard errors. Experiments were performed for three times.

CellTiter-Blue assay was employed to evaluate the cell viability in response to MWCNTs. S-MWCNTs, when added to cell cultures of immune cells RAW264.7, did not elicited significant damage under almost all treatment conditions (Figure 4a). To compare the length effect of MWCNTs, cells were treated with L-MWCNTs under the same conditions. As shown in Figure 4a, L-MWCNTs induced significant toxic effects in RAW264.7 cells at higher concentrations (200 μg/mL) and longer incubation (>72 h).

Carbon nanotubes and other nano-structures by virtue of their surface energy and reactivity pose special problems in the evaluation of their toxicity, not encountered in assessment of standard cytotoxicity studies for drugs. MWCNTs are significantly bigger and have more complex structure than most cytotoxic drugs, and many unknown factors may influence tests used to determine their cytotoxicity. In order to validate the cell viability data obtained by CellTiter-Blue, we used a second assay, trypan blue exclusion. As shown in Figure 4b, trypan blue manual counting revealed overall lower cell survival rates of RAW264.7 cells in response to short and long MWCNTs. Although no significant toxicity was detected by CellTiter-Blue assay for S-MWCNTs to RAW264.7 cells, trypan blue counting, however, demonstrated remarkably lower viability by S-MWCNTs than control at concentrations at 50 μg/mL and above (Figure 4b). The length effect of MWCNTs evaluated by the second assay was detected earlier (at 24 h) by trypan blue counting than revealed by the CellTiter-Blue assay. These results indicate that standard cell viability assays like the CellTiter-Blue assay may give inaccurate or even false information for the assessment of MWCNT toxicity, and short MWCNTs are although safe for use in general with most cells, they can still cause damage to certain immune cell types.

**Figure 5:**
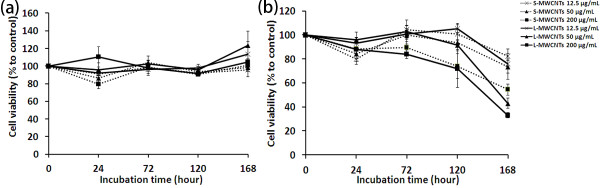
**Cell viability of MCF-7 cells.** Measured by (**a**) CellTiter-Blue assay and (**b**) trypan blue counting in response to different concentrations and incubation times of S- and L-MWCNTs. Cells were treated as described in the ‘Methods’ section, and data were expressed as mean ± standard errors. Experiments were performed for three times.

The data from MCF-7, again suggest the unsuitability of conventional cytotoxicity assays for nanotoxicity evaluation (Figure 5), showed that overall toxicity of MWCNTs to these cells under most experimental conditions was smaller than that to RAW264.7 cells especially at higher doses (Figure 5), in which both S- and L-MWCNTs were shown to damage immune cells even at early stage of incubation (Figure 4b). Length-dependent toxicity of MWCNTs to MCF-7 cells was also observed at later stage of incubation (7 days), which was distinct from that of RAW264.7 cells. These data demonstrated that RAW264.7 cells are more vulnerable to MWCNT encounter than MCF-7 cells under the experimental conditions.

### ELISA measurements of cytokine secretion of RAW264.7

**Figure 6:**
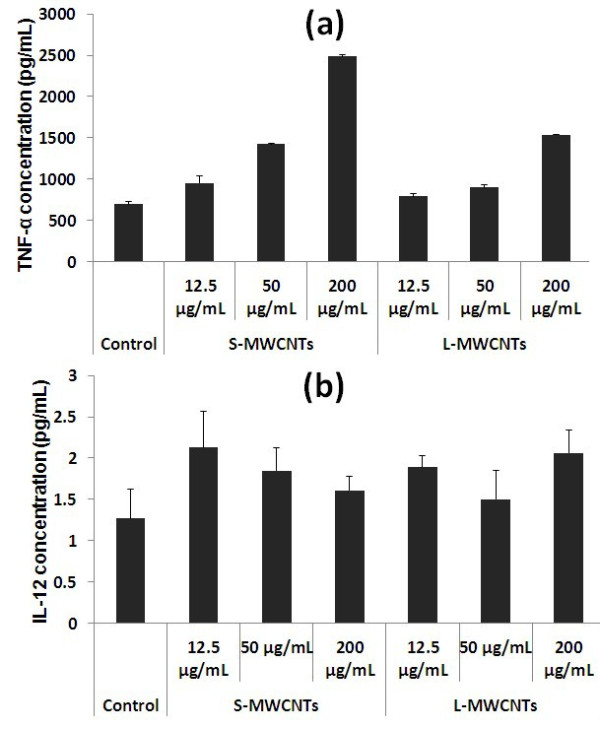
**TNF-α (a) and IL-12 (b) released in culture media by RAW264.7 cells.** Experiments were performed in triplicates for three times.

Based on the viability data showing that RAW264.7 cells were more susceptible to damage by MWCNTs than MCF-7 cells, the underlying mechanism such as release of pro-inflammatory cytokine by immune cells in response to MWCNTs [16] was studied. TNF-α is one of the major cytokines secreted by immune cells under stress conditions [5]. Hence, we investigated the release of TNF-α to culture media by RAW264.7 upon MWCNT treatment. As shown in Figure 6a, S-MWCNTs stimulated RAW264.7 cells to produce higher levels of TNF-α than L-MWCNTs in a dose-dependent manner (up to 2,486.4 and 1,537 pg/mL at concentration of 200 μg/mL by S- and L-MWCNTs, respectively). These results implicate the ability of TNF-α induction by MWCNTs in macrophages. IL-12 is an inflammatory mediator released by dendritic cells and macrophages in response to cytotoxic foreign bodies. As shown in Figure 6b, only a small increase on the release of IL-12 by RAW264.7 when exposed to S- and L-MWCNTs for 24 h was observed, and the level of released cytokine was similar in both treatments.

### ROS production of cells in response to MWCNTs

**Figure 7:**
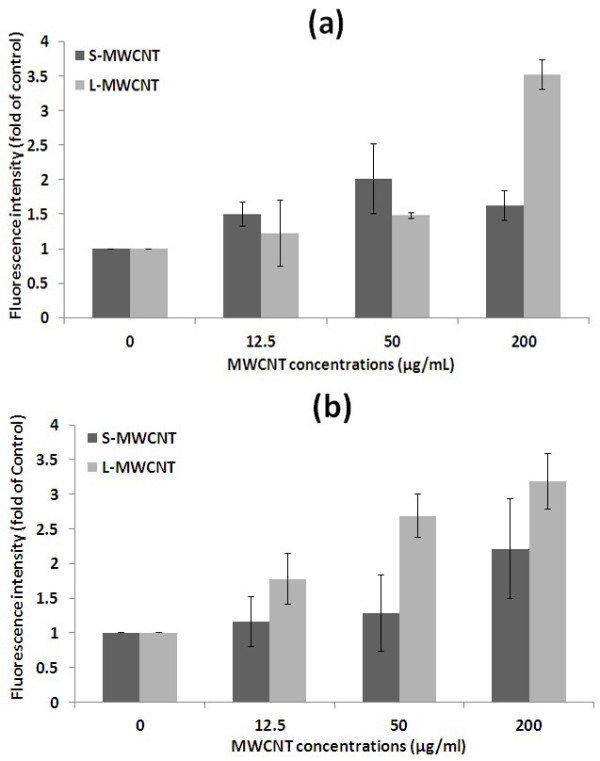
**ROS production.** By (**a**) RAW264.7 and (**b**) MCF-7 cells in response to MWCNTs is dose and length dependent. Experiments were performed in duplicates for three times.

The effect of S- and L-MWCNTs on cellular oxidative stress was assessed by the formation of intracellular ROS since the generation of ROS by nanomaterials has been linked to a general toxic response [17]. As shown in Figure 7, S- and L-MWCNTs induced the formation of ROS in both cell lines in a dose- and length-dependent manner. L-MWCNTs at 200 μg/ml induced much higher level of ROS in RAW264.7 cells than S-MWCNTs after 24-h incubation in agreement with the cell viability data in which L-MWCNTs exhibited significantly higher toxicity than S-MWCNTs to these cells at earlier stage of incubation (Figure 4b). In MCF-7 cells, L-MWCNTs showed greater toxicity than S-MWCNT, causing significant increase in ROS (*p* < 0.05) at all tested concentrations after 24-h incubation (Figure 7b). Although the data in cell oxidative stress are partially in agreement with that obtained by standard endpoint cell viability assays, ROS measurement revealed earlier cell response (24-h incubation) that could not be detected by cell viability assays (Figures 4b and 5b), suggesting MWCNTs' cytotoxicity should be evaluated by analysing different aspects of cell response so as to get improved safety profile of this nanomaterials.

## Discussions

There is evidence that the risk of MWCNT nanotoxicity increases with their length. Our study showed that highly purified, well-dispersed MWCNTs exhibit different degree of toxicity depending on their length as well as cell types tested. In addition, the experimental design and methods used in the present study provide valuable information for interpretation of cell toxicity analysis and for risk evaluation in biomedical applications of MWCNTs.

In the evaluation of the MWCNT toxicity *in vitro*, choice of appropriate methods for cell viability is crucial in view of conflicting data in some of the reported studies, which can be explained by the various *in vitro* cell viability tests. In many of these studies, MTT assay has been used. Subsequently, a problem with the MTT assay was identified as the MTT-formazan crystals bind to the CNTs, accounting for the false positive results by this assay [18]. Other authors have reported interactions of CNTs with various indicator dyes: Commasie Blue, Alamar Blue (same as CellTiter-Blue), neutral red, MTT, and WST-1 (same as MTS) [19,20]. One report evaluating the interference of MWCNTs with resazurin confirmed a significant decrease in the signal (approximately 15 % to 20 %) during fluorescence detection in the presence of MWCNTs [21]. The present study also revealed similar interference between the CellTiter-Blue reagents and MWCNTs (data not shown). To minimize this effect in the CellTiter-Blue assays used in the present study, excessive MWCNTs in media were thoroughly washed and removed before adding the reagents. However, this failed to remove most MWCNTs attached to cell membranes when checked with microscopy. Therefore, it seems likely that during CellTiter-Blue assay the interference between MWCNTs (both intracellular and membrane bound) and assay reagents persists and could contribute to the false high cell viability results when validated by manual trypan blue counting. More complex mechanisms such as cell membrane-MWCNT interaction may also be involved in determining cellular responses to MWCNTs [22], all of which merit detailed further investigation.

As the immune system plays a key role in the protection against foreign materials, studies of MWCNT toxicity to immune cells are essential for *in vivo* translation of therapies based on MWCNTs. In this respect, tissue and circulating macrophages constitute an important component of the effector immune system responsible for the ingestion and clearance of particulate foreign bodies and, thus, deserve attention in nano-toxicity studies. Our data suggest that precautions are needed even when using short MWCNTs.

To study the possible mechanisms underlying the difference in their biocompatibility, S- and L-MWCNTs cellular uptake was investigated. RAW264.7 cells internalised more MWCNTs than the MCF-7 cells, partially explaining the difference in cytotoxicity by MWCNTs in two cell types, as the larger load of internalised MWCNTs may be responsible for increased disruption of the integrity of cell membrane and intracellular organelles. Cellular uptake cannot, however, be the only factor determining cell viability. As MWCNT-cell membrane interaction could affect cellular response and, therefore, cytotoxicity, the higher toxicity of L-MWCNTs may also be explained by such mechanism, which is yet to be clarified. It has been shown that macrophages experience difficulty in engulfing long CNTs, and incomplete internalisation or simply binding of MWCNTs by macrophages could impair the plasma membrane [23]. Additionally, membrane damage caused by carbon-based nanomaterials can be also ascribed to lipid peroxidation following contact between the nanomaterial and cell membrane [22].

Pro-inflammatory cytokine secretion is an important parameter in inducing apoptosis of immune cells as well as an indicator of the inflammatory response. Our studies have shown that in response to MWCNT treatment, RAW264.7 cells secreted considerable amount of TNF-α, indicative of the inflammatory response by these cells. However, in contrast to the cytotoxicity, S-MWCNTs induced more TNF-α production than L-MWCNTs. This finding is in agreement with the previous report by Brown et al. [16] that straighter and shorter nanotubes induced higher TNF-α production compared with other CNT samples. Since our viability data showed that L-MWCNTs were more cytotoxic to RAW264.7 cells, other mechanisms are probably involved, e.g. membrane-nanotube contact causing more cell membrane and intracellular organelle damage, lipid peroxidation, incomplete uptake of L-MWCNT, etc. IL-12 production by RAW264.7 in response to both types of MWCNTs was, however, minimal during 24-h incubation. Nevertheless, we cannot exclude IL-12 release during longer incubation periods. In addition to pro-inflammatory cytokine secretion by RAW264.7 cells, ROS generation by both cell lines was employed in the present study to compare MWCNT toxicity. Although previously reported, ROS production induced by CNT exposure was considered to be caused by the metal impurities [8,24]; our data demonstrate that both S- and L-MWCNTs, even when highly purified, induced excessive of ROS production within 24 h in dose- and length-dependent manner, suggesting the nanoscale nature of the MWCNTs itself influences the oxidative stress response of cells.

The present study has produced evidence that the current panel of cell viability assays for nanomaterials is not sensitive enough for providing an accurate toxicity profile of nanomaterial. In the short term, it is recommended that *in vitro* assays of nanomaterial toxicity should be confirmed by trypan blue exclusion, but there is an urgent need for the development of improved testing-based label-free approaches for measuring nanotoxicity in living cells. These approaches are being explored in our laboratory.

## Conclusions

In conclusion, this study confirms the importance of length of polymer-coated MWCNTs in cell damage and biocompatibility. It also demonstrates that the nanotoxicity of MWCNTs is cell specific and that epithelial cancer cells are more resistant to cell damage than immune cells. The amount of MWCNTs phagocytised by immune cells as well as pro-inflammatory responses induced by MWCNTs may play an important role in determining the degree of cytotoxicity. Due to possible complex interactions between CNTs and cellular components and between CNTs and standard cell viability assay systems, CNT nanotoxicity data should be carefully interpreted. Finally, there is a need for methodological research aimed at development of ‘reagent free’ tests of nanotoxicity of living cells exposed to nanomaterials.

## Competing interests

The authors declare that they have no competing interests.

## Authors' contributions

DL carried out the experiments and wrote the paper. LW supervised the work and did major revision on the paper. ZW involved in supervision and discussion. AC provided essential experiment facilities and advice, revised the paper, and made the final editing of the paper. All authors read and approved the final manuscript.

## Authors' information

AC was DL's PhD supervisor and is the professor and chief scientific advisor of the Institute of Medical Science and Technology (IMSaT), University of Dundee.

## Supplementary Material

Additional file 1 **Figure S1. SEM image of PF-127 coating on MWCNTs [**[25-27]**].** Click here for file

## References

[B1] Iijima S Helical microtubules of graphitic carbon. Nature 1991 354 56 58

[B2] Bianco A Kostarelos K Prato M Applications of carbon nanotubes in drug delivery Curr Opin Chem Biol 2005 9 674 10.1016/j.cbpa.2005.10.005 16233988

[B3] Kam NWS O'Connell M Wisdom JA Dai H Carbon nanotubes as multifunctional biological transporters and near-infrared agents for selective cancer cell destruction Proc Natl Acad Sci 2005 102 11600 11605 10.1073/pnas.0502680102 16087878 PMC1187972

[B4] Giersig M Firkowska I Troszczynska J Rojas-Chapana J Cell manipulation and tissue engineering at the nanoscale NanoBiotechnology 2005 1 290 292 10.1007/s12030-005-0045-5

[B5] Sato Y Yokoyama A Shibata K-I Akimoto Y Ogino S-I Nodasaka Y Kohgo T Tamura K Akasaka T Uo M Motomiya K Jeyadevan B Ishiguro M Hatakeyama R Watari F Tohji K Influence of length on cytotoxicity of multi-walled carbon nanotubes against human acute monocytic leukemia cell line THP-1 in vitro and subcutaneous tissue of rats in vivo Mol Biosyst 2005 1 176 182 10.1039/b502429c 16880981

[B6] Vittorio O Raffa V Cuschieri A Influence of purity and surface oxidation on cytotoxicity of multiwalled carbon nanotubes with human neuroblastoma cells Nanomedicine: Nanotechnol, Biol Med 2009 5 424 431 10.1016/j.nano.2009.02.006 19341817

[B7] Sayes CM Liang F Hudson JL Mendez J Guo W Beach JM Moore VC Doyle CD West JL Billups WE Functionalization density dependence of single-walled carbon nanotubes cytotoxicity in vitro Toxicol Lett 2006 161 135 142 10.1016/j.toxlet.2005.08.011 16229976

[B8] Pulskamp K Diabat S Krug HF Carbon nanotubes show no sign of acute toxicity but induce intracellular reactive oxygen species in dependence on contaminants Toxicol Lett 2007 168 58 74 10.1016/j.toxlet.2006.11.001 17141434

[B9] VanHandel M Alizadeh D Zhang L Kateb B Bronikowski M Manohara H Badie B Selective uptake of multi-walled carbon nanotubes by tumor macrophages in a murine glioma model J Neuroimmunol 2009 208 3 9 10.1016/j.jneuroim.2008.12.006 19181390

[B10] Bianco A Hoebeke J Godefroy S Chaloin O Pantarotto D Briand J-P Muller S Prato M Partidos CD Cationic carbon nanotubes bind to CpG oligodeoxynucleotides and enhance their immunostimulatory properties J Am Chem Soc 2004 127 58 59 15631447 10.1021/ja044293y

[B11] Meng J Duan J Kong H Li L Wang C Xie S Chen S Gu N Xu H Yang X-D Carbon nanotubes conjugated to tumor lysate protein enhance the efficacy of an antitumor immunotherapy Small 2008 4 1364 1370 10.1002/smll.200701059 18720440

[B12] Bardi G Tognini P Ciofani G Raffa V Costa M Pizzorusso T Pluronic-coated carbon nanotubes do not induce degeneration of cortical neurons in vivo and in vitro Nanomedicine: Nanotechnol, Biol Med 2009 5 96 104 10.1016/j.nano.2008.06.008 18693142

[B13] Li ZF Luo GH Zhou WP Wei F Xiang R Liu YP The quantitative characterization of the concentration and dispersion of multi-walled carbon nanotubes in suspension by spectrophotometry Nanotechnology 2006 17 3692 3698 10.1088/0957-4484/17/15/012

[B14] Hirano S Fujitani Y Furuyama A Kanno S Uptake and cytotoxic effects of multi-walled carbon nanotubes in human bronchial epithelial cells Toxicol Appl Pharmacol 2010 249 8 15 10.1016/j.taap.2010.08.019 20800606

[B15] Raffa V Ciofani G Nitodas S Karachalios T D'Alessandro D Masini M Cuschieri A Can the properties of carbon nanotubes influence their internalization by living cells? Carbon 2008 46 1600 1610 10.1016/j.carbon.2008.06.053

[B16] Brown DM Kinloch IA Bangert U Windle AH Walter DM Walker GS Scotchford CA Donaldson K Stone V An in vitro study of the potential of carbon nanotubes and nanofibres to induce inflammatory mediators and frustrated phagocytosis Carbon 2007 45 1743 1756 10.1016/j.carbon.2007.05.011

[B17] Wani MY Hashim MA Nabi F Malik MA Nanotoxicity: dimensional and morphological concerns Adv Phys Chem 2011

[B18] Worle-Knirsch JM Pulskamp K Krug HF Oops they did it again! Carbon nanotubes hoax scientists in viability assays Nano Lett 2006 6 1261 1268 10.1021/nl060177c 16771591

[B19] Casey A Herzog E Davoren M Lyng FM Byrne HJ Chambers G Spectroscopic analysis confirms the interactions between single walled carbon nanotubes and various dyes commonly used to assess cytotoxicity Carbon 2007 45 1425 1432 10.1016/j.carbon.2007.03.033

[B20] Davoren M Herzog E Casey A Cottineau B Chambers G Byrne HJ Lyng FM In vitro toxicity evaluation of single walled carbon nanotubes on human A549 lung cells Toxicol Vitr 2007 21 438 448 10.1016/j.tiv.2006.10.007 17125965

[B21] Rotoli BM Bussolati O Bianchi MG Barilli A Balasubramanian C Bellucci S Bergamaschi E Non-functionalized multi-walled carbon nanotubes alter the paracellular permeability of human airway epithelial cells Toxicol Lett 2008 178 95 102 10.1016/j.toxlet.2008.02.007 18403140

[B22] Ding L Stilwell J Zhang T Elboudwarej O Jiang H Selegue JP Cooke PA Gray JW Chen FF Molecular characterization of the cytotoxic mechanism of multiwall carbon nanotubes and nano-onions on human skin fibroblast Nano Lett 2005 5 2448 2464 10.1021/nl051748o 16351195 PMC2733876

[B23] Hirano S Kanno S Furuyama A Multi-walled carbon nanotubes injure the plasma membrane of macrophages Toxicol Appl Pharmacol 2008 232 244 251 10.1016/j.taap.2008.06.016 18655803

[B24] Schrand AM Dai L Schlager JJ Hussain SM Osawa E Differential biocompatibility of carbon nanotubes and nanodiamonds Diamond Relat Mater 2007 16 2118 2123 10.1016/j.diamond.2007.07.020

[B25] Liu Z Tabakman S Welsher K Dai H Carbon nanotubes in biology and medicine: in vitro and in vivo detection, imaging and drug delivery Nano Res 2009 2 85 120 10.1007/s12274-009-9009-8 20174481 PMC2824900

[B26] Carrillo A Swartz JA Gamba JM Kane RS Chakrapani N Wei BQ Ajayan PM Noncovalent functionalization of graphite and carbon nanotubes with polymer multilayers and gold nanoparticles Nano Lett 2003 3 1437 1440 10.1021/nl034376x

[B27] O'Connell MJ Bachilo SM Huffman CB Moore VC Strano MS Haroz EH Rialon KL Boul PJ Noon WH Kittrell C Ma J Hauge RH Hauge RB Smalley RE Band gap fluorescence from individual single-walled carbon nanotubes Science 2002 297 593 596 10.1126/science.1072631 12142535

