# Supplementary material for: Different cellular response mechanisms contribute to the length-dependent cytotoxicity of multi-walled carbon nanotubes
Source: Nanoscale Res Lett. 2012 Jul 2;7(1):361. doi: 10.1186/1556-276X-7-361 (PMC3461426; doi:10.1186/1556-276X-7-361)
Supplement: Additional file 1 — Figure S1. SEM image of PF-127 coating on MWCNTs [[25-27]]. [file 1556-276X-7-361-S1.docx]

**Different cellular response mechanisms contribute to the length-dependent cytotoxicity of multi-walled carbon nanotubes**

**Supplementary Information**

Dun Liu, Lijun Wang, Zhigang Wang, and Alfred Cuschieri*

Institute for Medical Science and Technology, University of Dundee, Dundee Medipark, Dundee, UK

**Characterization of PF-127 coating by SEM**

Scanning electron microscopy (SEM) was employed to assess effective PF-127 coating on the MWCNTs. For this characterisation, samples of PF-127 coated MWCNTs (before resuspended in DMEM) were washed only once ddH_2_O by centrifugation at 40 000 g, 4^o^C, to remove the salts and excessive PF-127 in medium. A small drop (10 μL) of MWCNT solution (5 μg/mL) was placed onto an alumina substrate and allowed to dry at room temperature. Dried specimens were then coated with 8 nm Au/Pd using a Cressington 208HR sputter coater. Specimens were examined using a Philips XL30 ESEM operating at an accelerating voltage of 15 kV.

Unlike the well washed MWCNTs shown in Fig 1 in the main text, which have smooth surface after 8 times thoroughly wash, MWCNTs washed only once were covered by polymers, which are speculated to be PF-127 (Fig S1). Since PF-127 coating is not covalently bound to the CNTs, the carbon structures of MWCNTs were well preserved [1, 2], and the nanotubes have broad absorption spectra [3], the PF-127 coating on MWCNTs cannot be detected by Fourier transform infrared spectroscopy (FTIR) (data not shown).


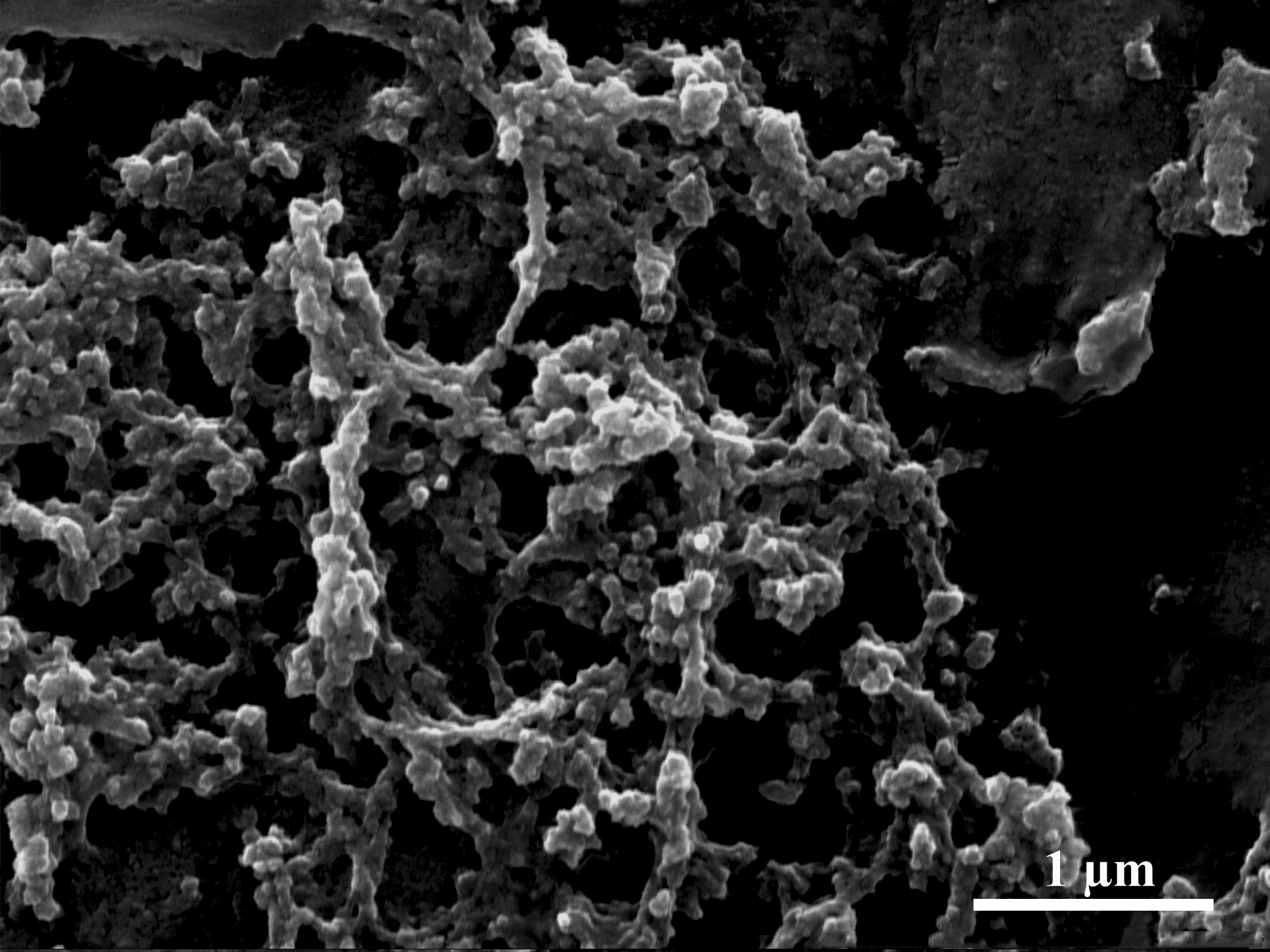


Figure S1. SEM image of PF-127 coating on MWCNTs.

1. Liu Z, Tabakman S, Welsher K, Dai H: **Carbon Nanotubes in Biology and Medicine: In vitro and in vivo Detection, Imaging and Drug Delivery.** *Nano Res* 2009, **2:**85-120.

2. Carrillo A, Swartz JA, Gamba JM, Kane RS, Chakrapani N, Wei BQ, Ajayan PM: **Noncovalent functionalization of graphite and carbon nanotubes with polymer multilayers and gold nanoparticles.** *Nano Letters* 2003, **3:**1437-1440.

3. O'Connell MJ, Bachilo SM, Huffman CB, Moore VC, Strano MS, Haroz EH, Rialon KL, Boul PJ, Noon WH, Kittrell C, et al: **Band Gap Fluorescence from Individual Single-Walled Carbon Nanotubes.** *Science* 2002, **297:**593-596.
